# Reply to Schouten et al. Comment on “Bilika et al. Applying Nociplastic Pain Criteria in Chronic Musculoskeletal Conditions: A Vignette Study. *J. Clin. Med.* 2025, *14*, 1179”

**DOI:** 10.3390/jcm15103741

**Published:** 2026-05-13

**Authors:** Paraskevi Bilika, Jo Nijs, Evdokia Billis, Zacharias Dimitriadis, Achilleas Paliouras, Konstantina Savvoulidou, Nikolaos Strimpakos, Eleni Kapreli

**Affiliations:** 1 Clinical Exercise Physiology and Rehabilitation Research Laboratory, Department of Physiotherapy, School of Health Sciences, University of Thessaly, 35100 Lamia, Greece; apaliouras@uth.gr (A.P.); ksavvoulidou@uth.gr (K.S.); ekapreli@uth.gr (E.K.); 2 Pain in Motion Research Group (PAIN), Department of Physiotherapy, Human Physiology and Anatomy, Faculty of Physical Education & Physiotherapy, Vrije Universiteit Brussel, 1090 Brussels, Belgium; jo.nijs@vub.ac.be; 3 Chronic Pain Rehabilitation, Department of Physical Medicine and Physiotherapy, University Hospital Brussels, 1090 Brussels, Belgium; 4 Unit of Physiotherapy, Department of Health and Rehabilitation, Institute of Neuroscience and Physiology, Sahlgrenska Academy, University of Gothenburg, SE-405 30 Gothenburg, Sweden; 5 Physiotherapy Department, School of Health Rehabilitation Sciences, University of Patras, 26504 Patras, Greece; billis@upatras.gr; 6 Health Assessment and Quality of Life Research Laboratory, Department of Physiotherapy, School of Health Sciences, University of Thessaly, 35100 Lamia, Greece; zdimitriadis@uth.gr (Z.D.); nikstrimp@uth.gr (N.S.); 7 Division of Musculoskeletal & Dermatological Sciences, University of Manchester, Manchester M13 9PL, UK

We thank the authors for their constructive comments on our work [1]. We appreciate their interest in our study and the opportunity to further clarify key methodological aspects that may support a more precise interpretation of our findings.

## 1. Comment 1: Assessment of Evoked Pain Hypersensitivity

We appreciate the important point raised regarding the fourth step of the IASP clinical grading system, namely the assessment of evoked pain hypersensitivity. As noted, this criterion relies on physical examination, which makes it difficult to apply in a vignette-based design.

In our study, information related to evoked pain hypersensitivity was incorporated into most vignettes through descriptions such as sensitivity to palpation or movement-evoked pain. However, we acknowledge that this information was not uniformly or explicitly presented across all cases, including the example vignette no. 20, in Supplementary Material S1. In these cases, raters based their judgments on the available clinical information and treated the lack of clear findings as the feature not being present, following the assessment instructions.

Importantly, according to the IASP clinical criteria and grading algorithm, the evaluation of evoked pain hypersensitivity is not required when there is strong clinical evidence supporting alternative pain mechanisms (e.g., nociceptive or neuropathic pain). Therefore, in vignettes representing nociceptive pain, the absence of sensory testing information reflects both the structure of the clinical algorithm and real-world clinical reasoning, where such assessment may not be performed when another mechanism is sufficiently supported.

We agree that variability in the reporting of this feature may influence the consistency with which this criterion was applied and may have contributed to the high inter-rater reliability observed. More broadly, this reflects a known limitation of vignette-based methodologies when attempting to operationalise clinical criteria that require physical examination [2,3]. Future studies that include real clinical assessments and sensory testing procedures will allow a more robust evaluation of this component. Accordingly, the assessment of evoked pain hypersensitivity in this context should be interpreted with due consideration of these methodological constraints.

## 2. Comment 2: Criterion Validity and Independence of the Reference Standard

This important methodological point is well taken. As acknowledged in our study, in the absence of an established gold standard for nociplastic pain, the use of an expert panel as a reference standard carries inherent limitations.

At the same time, the use of expert consensus as a reference standard is common in diagnostic research, particularly in fields where no objective gold standard exists. For example, Bertens et al. [4] showed that expert panel diagnosis is frequently used in areas such as psychiatry and cardiovascular disease, where diagnostic constructs are complex and not directly measurable. Similarly, Rutjes et al. [5] describe consensus-based reference standards as a pragmatic and promising approach in such contexts, although they require careful interpretation and transparent reporting.

As noted, both the index test (IASP clinical criteria) and the reference standard (expert-based classification) are grounded in evidence-informed constructs, including widespread pain, exclusion of nociceptive and neuropathic mechanisms, and features of pain hypersensitivity. This conceptual overlap may potentially inflate estimates of diagnostic agreement.

Importantly, the vignettes were constructed with a pre-defined (a priori) pain mechanism. The expert-based classification was performed prior to the publication and clinical implementation of the IASP criteria and grading system. Therefore, the experts were not informed by, nor did they apply, the IASP criteria during their evaluations. Instead, their judgements were based on the contemporary literature and clinical reasoning available at that time, which already included efforts to identify nociplastic pain (or central sensitisation-related pain) as distinct from nociceptive and neuropathic pain. Such approaches have been described in earlier work, including studies by Smart et al. [6] and Nijs et al. [7].

In addition, the reference standard was based on expert clinical judgement rather than a structured or algorithm-driven approach, whereas the IASP criteria represent a formalised and standardised decision-making framework. This distinction supports a degree of methodological independence between the two approaches, although not full conceptual independence.

Overall, our approach aligns with pragmatic diagnostic accuracy studies, where less-than-perfect reference standards are used to inform clinical decision-making [8]. However, we agree that the term “criterion validity” may overstate the strength of inference in this context.

These comments provide useful insight and support a more nuanced interpretation of our findings. We agree that they highlight important methodological challenges in the evaluation of nociplastic pain and reinforce the need for further research in this area.
